# Ocular Blood Flow in Rabbits under Deep Anesthesia: A Real-Time Measurement Technique and Its Application in Characterizing Retinal Ischemia

**DOI:** 10.1038/s41598-018-24141-4

**Published:** 2018-04-09

**Authors:** Mehwish Saba Bhatti, Tong Boon Tang, Hui Cheng Chen

**Affiliations:** 1 Centre for Intelligent Signal and Imaging Research (CISIR), Department of Electrical & Electronic Engineering, Universiti Teknologi PETRONAS, Bandar Seri Iskandar, 32610 Perak, Malaysia; 2 Department of Companion Animal Medicine and Surgery, Faculty of Veterinary Medicine, Universiti Putra Malaysia, 43400 UPM Serdang, Malaysia

## Abstract

In this study, we reported a new technique based on laser speckle flowgraphy to record the ocular blood flow in rabbits under deep anesthesia, and proposed parameters to characterize retinal ischemia. We applied the proposed technique to study the correlation of blood flow between the eyes of normal non-anesthetized animals, and to characterize the occlusion of the internal carotid artery (ICA) and external carotid artery (ECA). We established a correlation in blood flow between the eyes of non-anesthetized animals, and derived two new parameters, namely, the laterality index and vascular perfusion estimate (VPE). Our experimental results from 16 eyes (of 13 New Zealand white rabbits) showed a reduction in ocular blood flow with a significant decrease in the VPE after the occlusion of the ECA (*p* < 0.001). A low/minimal effect on blood flow was observed with the occlusion of the ICA. In conclusion, we demonstrated a means for the real-time measurement of the ocular blood flow in rabbits under deep anesthesia by using laser speckle flowgraphy and the VPE as an indicator of successful occlusion. The proposed technique might be applicable in quantifying the efficacy of new drugs and interventions for the treatment of retinal ischemia.

## Introduction

Atherosclerosis of the carotid artery, which often occurs at the bifurcation node of the external carotid artery (ECA) and internal carotid artery (ICA), may lead to retinal ischemia or retinal hypoperfusion^1–3^. Retinal ischemia may present as ischemic optic neuropathy, obstructive or diabetic retinopathy, amaurosis fugax, or neovascular or rubeotic glaucoma^4^. Retinal ischemia often starts with atherosclerosis of the carotid artery^5^, and subsequently hypoperfusion of the ocular tissues due to a decrease in blood pressure in the ophthalmic artery. A previous study reported that about one-third of the patients with carotid artery disease developed ocular ischemic syndrome (OIS)^6^. Chronic and advanced OIS can remain asymptomatic, and may pose a threat to visual acuity if left undiagnosed^7^. About 91% of the eyes in patients with carotid artery disease has been reported to experience a decrease in visual acuity^8^.

Animal models of retinal ischemia are indispensable in basic and applied research for the development of new therapeutic approaches and treatments of human disease. Retinal ischemia can be induced in many ways, such as by increasing the intraocular pressure, cerebral artery occlusion, chronic carotid ligation, photocoagulation of the retinal vessels, central retinal artery (CRA) occlusion, optic nerve bundle ligation, and endothelin administration^9^. Models of cerebral ischemia requiring ligation or transection of the common carotid artery (CCA) or ECA have also been shown to develop retinal ischemia. These models exhibit deterioration of the systemic conditions, which in return leads to changes in ocular perfusion^7,10^.

Animal models of retinal ischemia have been widely used as vehicles to assess the efficacy and adverse effects of new drugs. One example is the murine model of retinal ischemia developed by Lelong *et al*. in which retinal ischemia was induced by occluding both the pterygopalatine artery and ECA^11^. Ogishima *et al*. used the model to examine the efficacy of the drug edaravone, a free radical scavenger, for the treatment of retinal ischemia^6^. Dittmar *et al*. showed that retinal ischemia may be induced in rats via a commonly used endovascular filament at the middle cerebral artery (MCA); however, this also caused additional damage to the neural tissues that might affect the actual outcome of retinal ischemia^10^. The temporal dynamics of ocular blood flow associated with the occlusion of two major branches of the CCAs, the ECA and ICA, still remain unknown. We argue that the temporal dynamics might provide an insight into the characteristics of retinal ischemia. New parameters are needed to characterize perfusion to detect the early signs of hypoperfusion, for instance. These parameters can also be used to quantify the effects of various interventions on treating retinal hypoperfusion or ischemia.

The well-established rabbit models of cerebral ischemia often require ligation or transection of the ECA, e.g., the cerebral ischemia model developed by Yang *et al*., which requires the transection of the ECA to enter a guide wire into the ICA^12^, or the embolus model of cerebral ischemia developed by Lapchak *et al*., which requires the ligation of the ECA to prevent any blood clot from entering the ECA^13^. These models, in addition to causing cerebral ischemia, may be causing retinal ischemia when transecting or ligating the ECA. This study aimed to investigate how to assess dynamic changes in ocular blood flow before, during, and after the occlusion of the ECA and/or ICA in rabbits. We conducted this study using two separate experiments. The first experiment evaluated the correlation of blood flow in both the eyes. This experiment aimed to determine the relationship of blood flow between the eyes of non-anesthetized animals, and to derive new parameters/biomarkers, i.e., the laterality index (LI) and vascular perfusion estimate (VPE). The standard values of these two parameters were determined as the reference to differentiate between normal eyes and eyes with major occlusion in the carotid-ophthalmic pathway. In the second experiment, we examined ocular blood flow changes before, during, and after retinal ischemia in one eye of each anesthetized animal. The aim of the second experiment was to demonstrate that major occlusion in the carotid-ophthalmic pathway may be instantaneously detected using our new parameters (LI and VPE) by employing laser speckle flowgraphy (LSFG). All the experiments were carried out using a LSFG-NAVI system (Softcare, Fukuoka, Japan) for rabbits.

## Results

### Correlation of blood flow in both the eyes

Inducing retinal ischemia in one eye was expected to disrupt the symmetry of blood flow in both the eyes. We hypothesized that the symmetry of blood flow in both the eyes might be a good parameter to characterize retinal ischemia. However, a variation between the blood flows is inevitably present. To better understand it, data from both the eyes of non-anesthetized animals were obtained and categorized into two types, i.e., data from the eye with a higher mean blur rate (MBR) value and data from the eye with a lower MBR value. This was done because it has been observed that blood flow in both the eyes was mismatched and one eye had slightly higher MBR values than did the contralateral eye. The LSFG-NAVI software was used to segment the data into the vascular and tissue areas by manually setting the threshold value of MBR to 15. The MBR value for the vascular region alone was marked as MBR-Vascular or MV, and the MBR for the tissue area was marked as MBR-Tissue or MT. The MBR for the whole image was marked as MBR-All or MA. The difference in the mean MBR value of the vascular region and the mean MBR of the tissue region was marked as MV-MT. Scatter plots were used to demonstrate the correlation between the two groups, i.e., higher MBR values vs. lower MBR values for MV, MT, MA, and MV-MT. Linear regression was used to determine the correlation. We considered p-values less than 0.05 to be significant.

Previous studies have evaluated the symmetry of ocular blood flow in both the eyes. Aizawa *et al*. defined the ratio of the affected eye to the contralateral eye as a metric to determine the extent of detachment in patients with rhegmatogenous retinal detachment^14^. Similarly, Shinohara *et al*. defined the LI as a ratio of the MBR value of the occluded eye to that of the non-occluded eye to diagnose patients with OIS^15^. In this study, we defined the LI as a ratio of the lower MBR value to the higher MBR value for normal eyes. The LI was calculated for all the parameters, namely, MV, MT, MA, and MV-MT. We then computed the standard LI value (*S*_*LI*_) by using the following expression:

1 $${S}_{LI}={\mu }_{LI}\pm {\sigma }_{LI}$$

where *µ*_*LI*_ is the mean value obtained from the whole population, and *σ*_*LI*_ is its standard deviation.

**Figure 1:**
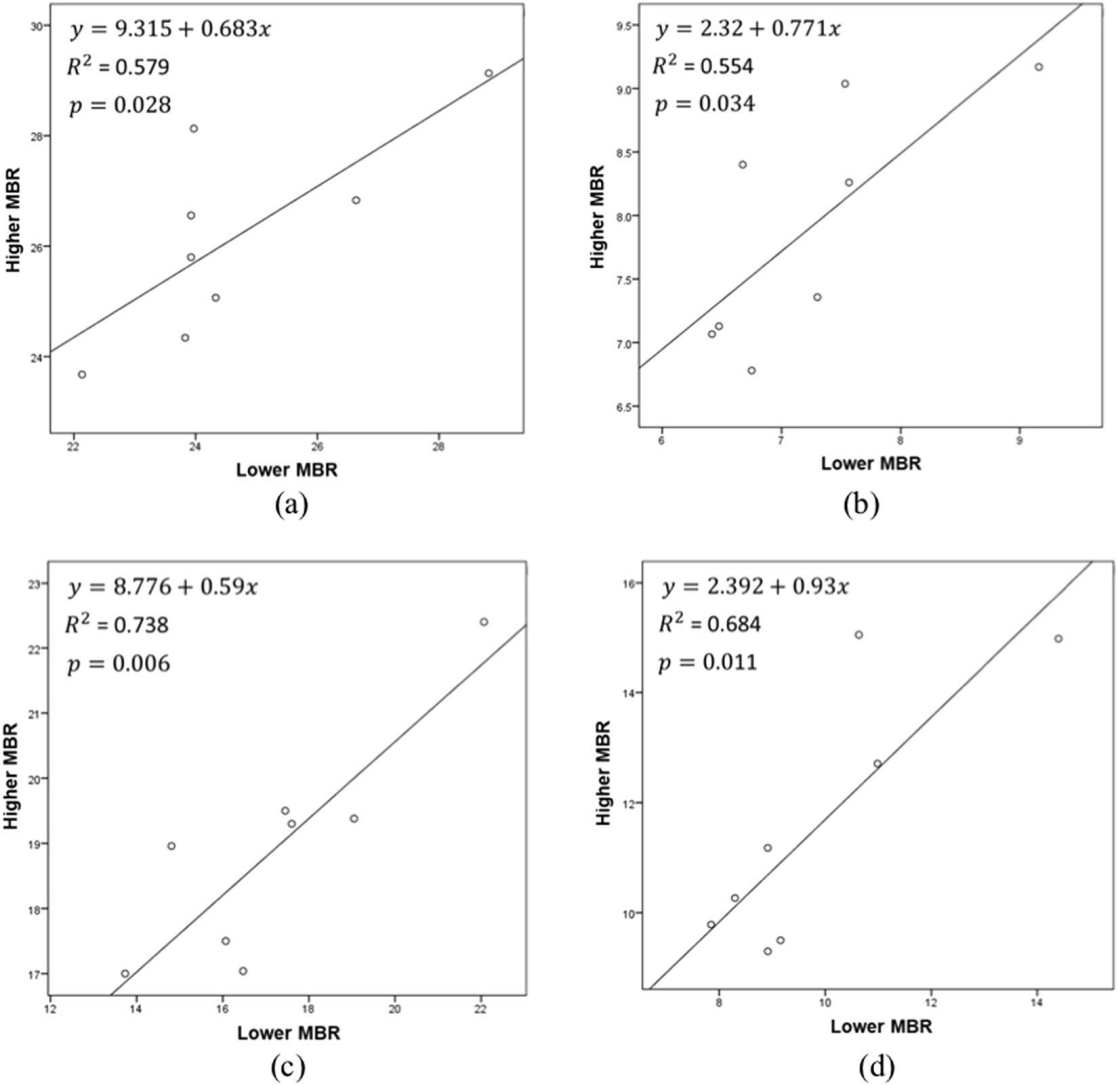
Scatter plots of eyes with lower MBR against contralateral eyes with high MBR—(**a**) mean value of the vascular region (MV), (**b**) mean value of the tissue region (MT), (**c**) mean value of the entire image (MA), and (**d**) difference of the mean value of the vascular region from the mean value of the tissue region (MV-MT).

In normal healthy rabbits, the false color maps obtained using the LSFG-NAVI software for both the eyes were almost identical. The mean MBR value in the eyes with the lower MBR value was 24.70 ± 2.07 for the vascular region (MV), 7.23 ± 0.90 for the tissue region (MT), 9.90 ± 2.11 for the entire image (MA), and 17.16 ± 2.59 for MV-MT. The mean MBR value in the eyes with the higher MBR value was 26.19 ± 1.86 for only the vascular region (MV), 7.90 ± 0.94 for the tissue region (MT), 11.60 ± 2.37 for the entire image (MA), and 18.89 ± 1.77 for MV-MT. All the parameters, namely, MV, MT, MA, and MV-MT, were compared for both the eyes, and no significant difference was found for any of the regions (Student’s paired t-test). Based on our results, we found the standard LI value for MV to be 0.94 ± 0.5, MT to be 0.92 ± 0.08, MA to 0.86 ± 0.10, and MV-MT to be 0.91 ± 0.08. Supplementary Table S1 shows in detail the values of the mean, standard deviations, and limits of the *S*_*LI*_. The smallest standard deviation was found in MV. The experimental results further showed that the MBR values of the eye with higher MBR values correlated to that of the contralateral eye with lower MBR values, and this correlation was the highest in MA (R^2^ = 0.738; p < 0.006), as shown in Fig. 1.

We further defined a new parameter and named it the VPE. This parameter is a product of two other parameters, namely, the mean MBR value of the vascular region of the whole image data (MV) and the area ratio of blood stream in the whole image (ARBS). In this case, the ARBS is defined as the percentage of retinal vessel area within the optic nerve head (ONH)^16^. The standard value of VPE is given as follows:

2 $$VPE=ARBS\times MV$$

The VPE for the normal non-anesthetized animal was 461.95 ± 236.28.

### Ocular blood flow changes before, during, and after occlusion

**Figure 2:**
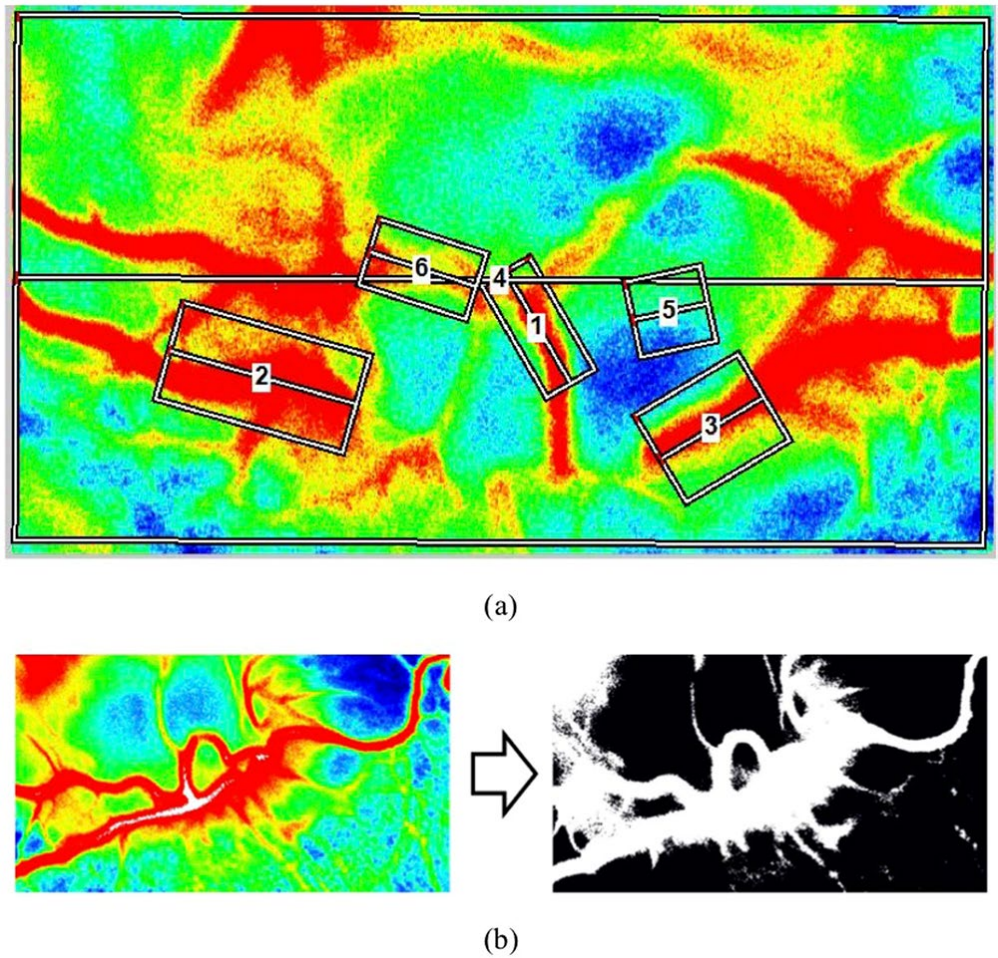
(**a**) Selected regions of interest—1, 2, 3, and 6 are vascular regions; 5 is a low-perfusion tissue region; and 4 is the entire image. (**b**) False color map and segmented image of the rabbit’s ONH.

The data were analyzed by selecting various regions of interest, as shown in Fig. 2(a). First, small regions of the major vessels entering the ONH were selected as the regions of interest (labeled as “vessel”). These included regions 1, 2, 3, and 6. Second, small regions of low-perfusion areas or tissue areas such as region 5 were identified (labeled as “tissue”). Third, the entire image, i.e., region 4, was segmented into the vascular and tissue areas, and was labeled as MV for vascular area, MT for tissue area, MA for the whole area, and MV-MT as the difference between the mean MBR values of the vascular and tissue areas. The vessel-tissue segmentation was performed using the LSFG-NAVI software, and the threshold value for segmentation was set to be MBR = 15. The result of segmentation is shown in Fig. 2(b). Finally, the whole ONH of the rabbit’s eye was selected as another region of interest as shown in Supplementary Figure S1. The results of the ONH area are shown in Supplementary Figure S2.

**Figure 3:**
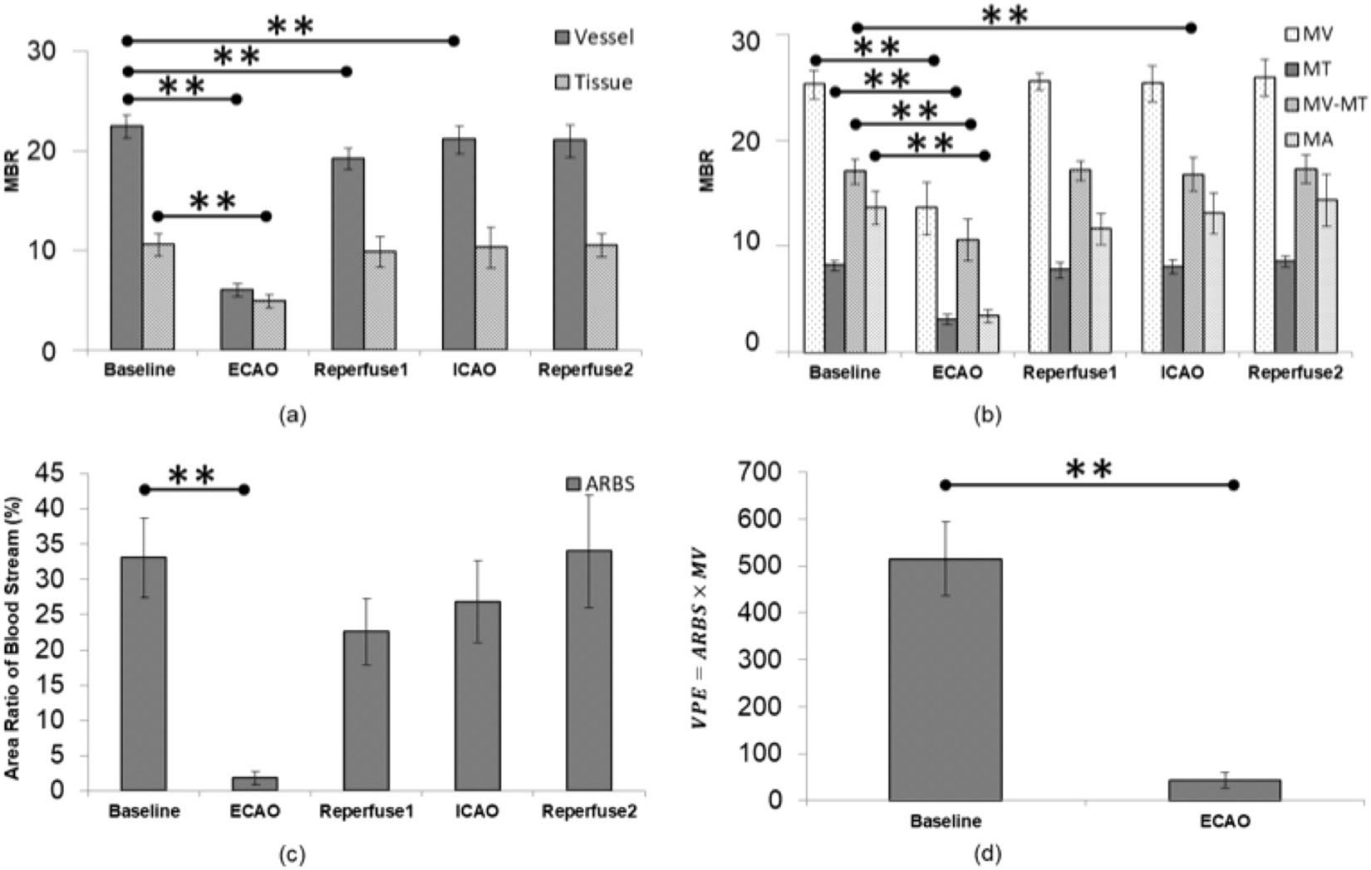
(**a**) The mean MBR during external carotid artery occlusion (ECAO), reperfusion after ECAO (Reperfuse1), internal carotid artery occlusion (ICAO), and reperfusion after ICAO (Reperfuse2) in the vascular and tissue regions of interest. (**b**) The mean MBR during ECAO, reperfusion after ECAO, ICAO, and reperfusion after ICAO of the vascular area of the entire image (MV), the tissue area of the entire image (MT), the whole area of the entire image (MA), and the mean MBR of the vascular region subtracted from the mean MBR value of the tissue region (MV-MT). (**c**) The area ratio of blood stream during ECAO, reperfusion after ECAO, ICAO, and reperfusion after ICAO in the entire image. (**d**) The vascular perfusion estimate (i.e., the product of the area ratio of blood stream and the mean MBR of the vascular region) during baseline recording and ECAO. Error bars represent the standard error of the mean. **Shows p < 0.001.

In total, 56 vessels were identified from the 16 (13 left and 3 right) eyes of the 13 New Zealand white rabbits. Data measured at the aforementioned small regions of “vessel” are shown in Fig. 3(a). The mean MBR value was 22.43 ± 8.61 as baseline, 6.00 ± 4.98 during ECA occlusion (ECAO), 19.18 ± 6.70 during reperfusion after ECAO (marked as “Reperfuse1”), 21.12 ± 7.94 during ICA occlusion (ICAO), and 20.98 ± 7.18 during reperfusion after ICAO (marked as “Reperfuse2”). The “vessel” data showed a significant decrease (p < 0.001) from baseline during ECAO, reperfusion after ECAO, and ICAO. Low-perfusion “tissue” was also analyzed. Their data are shown in Fig. 3(a). The mean MBR values in the low-perfusion “tissue” areas were 10.53 ± 5.01 as baseline, 4.90 ± 3.07 during ECAO, 9.82 ± 5.08 during reperfusion after ECAO, 10.30 ± 6.12 during ICAO, and 10.49 ± 2.62 during reperfusion after ICAO. The decrease in blood flow volume during ECAO was significant in the low-perfusion tissue areas (p < 0.001).

The entire image was also selected as another region of interest. The results of this region of interest are shown in Fig. 3(b–d). Figure 3(b) shows the MBR values during ECAO, reperfusion after ECAO (“Reperfuse1”), ICAO, and reperfusion after ICAO (“Reperfuse2”). Significant decrease in the MBR values for all the parameters, namely, MV, MT, MA, and MV-MT, was observed during ECAO than at baseline. Significant decrease during ICAO from the baseline was also observed for the parameter MV-MT. The ARBS was also calculated, and is shown in Fig. 3(c). A significant decrease was observed in the ARBS after ECAO. The VPE was calculated for non-occluded eyes (baselines) and the eyes undergoing ECAO. The standard VPE value was 515.25 ± 353.64 for normal eyes and 43.11 ± 54.34 for the eyes undergoing ECAO, as illustrated in Fig. 3(d). The mean MBR values from the entire image for normal non-anesthetized animals in experiment 2 and the baseline values before occlusion from the entire image for anesthetized animals in experiment 3 were not found to be significantly different (p < 0.05).

**Figure 4:**
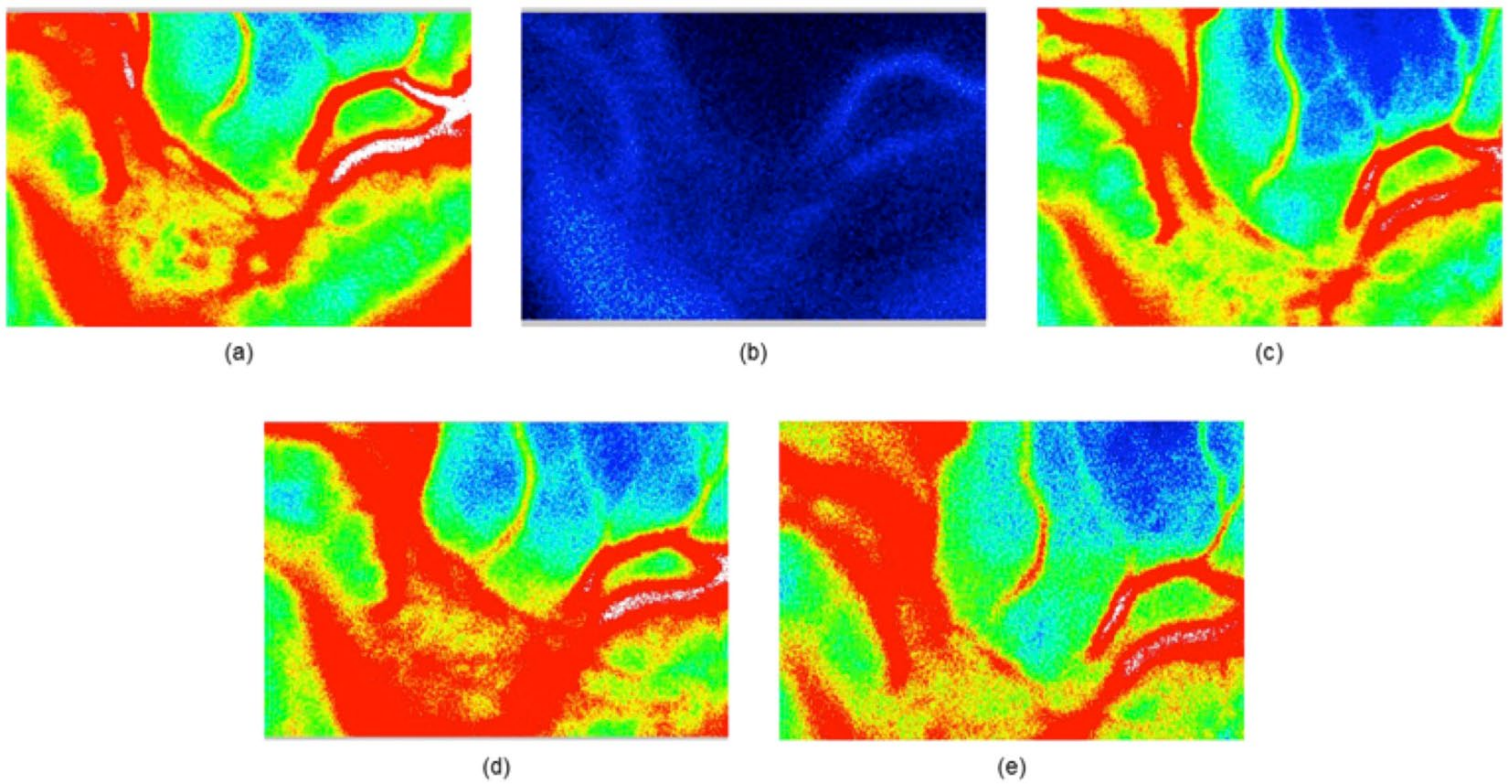
Effect of occlusions on ocular blood flow—(**a**) normal blood flow, (**b**) direct consequence of the 30-minute occlusion of the external carotid artery using a clip, (**c**) perfusion after the clip was removed, (**d**) during the 10-minute ICA occlusion, and (**e**) reperfusion after the ICA was unclipped.

The effects of occlusion of the two main branches, ECA and ICA, are illustrated in Fig. 4. Figure 4(a) shows normal blood flow and Fig. 4(b) shows the blood flow values plummeting to almost no perfusion as a direct consequence of ECAO. Perfusion was restored as soon as the clip occluding the ECA was removed, as shown in Fig. 4(c). ICAO for 10 minutes did not have any significant effect on ocular blood flow in any of the rabbits except for one, as shown in Fig. 4(d). The rabbit that showed a decrease in perfusion with ICAO also responded to ECAO. The baseline VPE of this rabbit was 515.7. The VPE after 15 minutes of ECAO and after reperfusion were 182.0 and 537.0, respectively. The VPE after 15 minutes of ICAO and after reperfusion were 98.7 and 780.2, respectively. For other rabbits, the reperfusion after ICAO showed a normal false color map, as shown in Fig. 4(e).

**Figure 5:**
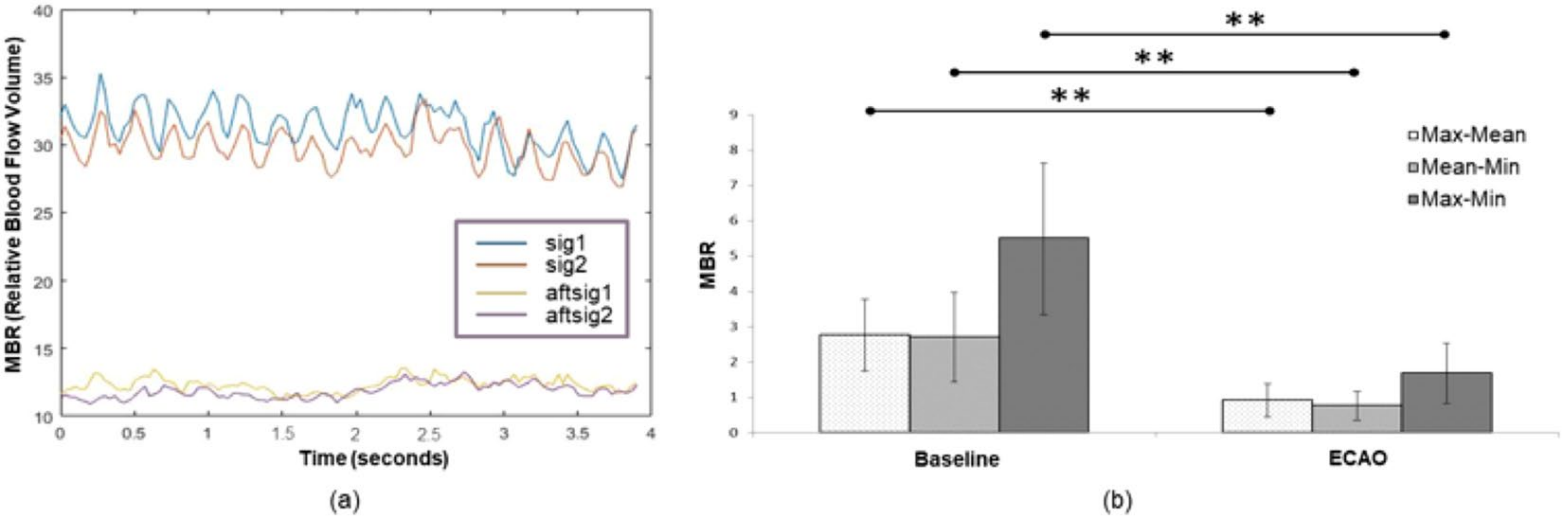
The effects of ECAO on blood flow oscillations, where (**a**) shows the changes in MBR amplitude and oscillating pattern. Sig1 and sig2 refer to the blood flow waveforms of two particular vascular regions before ECAO. Aftsig1 and aftsig2 refer to the blood flow waveforms sampled at the same vascular regions after ECAO; (**b**) shows different deviations of the MBR parameters, namely, Max-Mean, Mean-Min, and Max-Min before and after ECAO. Error bars show the standard error of the mean. **Shows p < 0.001.

Figure 5(a) shows ocular blood flow waveforms (normal and occluded eyes) measured using LSFG over a period of 4 seconds. ECAO had a noticeable effect on these oscillations. The effect was quantified by using three parameters, i.e., (i) the difference between the peak magnitude and the mean value of the waveform labeled as “Max-Mean,” (ii) the difference between the mean value and the minimum value of the blood flow waveform labeled as “Mean-Min,” and (iii) the difference between the maximum and the minimum values of blood flow waveform labeled as “Max-Min.” The significance of the results was calculated relative to the baseline, and results with p < 0.05 were considered significant. Figure 5(b) shows the parameter Max-Mean having a mean value of 2.77 ± 1.02 before occlusion and 0.92 ± 0.47 after ECAO; the parameter Mean-Min was 2.71 ± 1.27 before occlusion and 0.77 ± 0.41 after occlusion; and the parameter Max-Mean was 5.49 ± 2.15 before ECAO and 1.69 ± 0.85 after ECAO.

**Figure 6:**
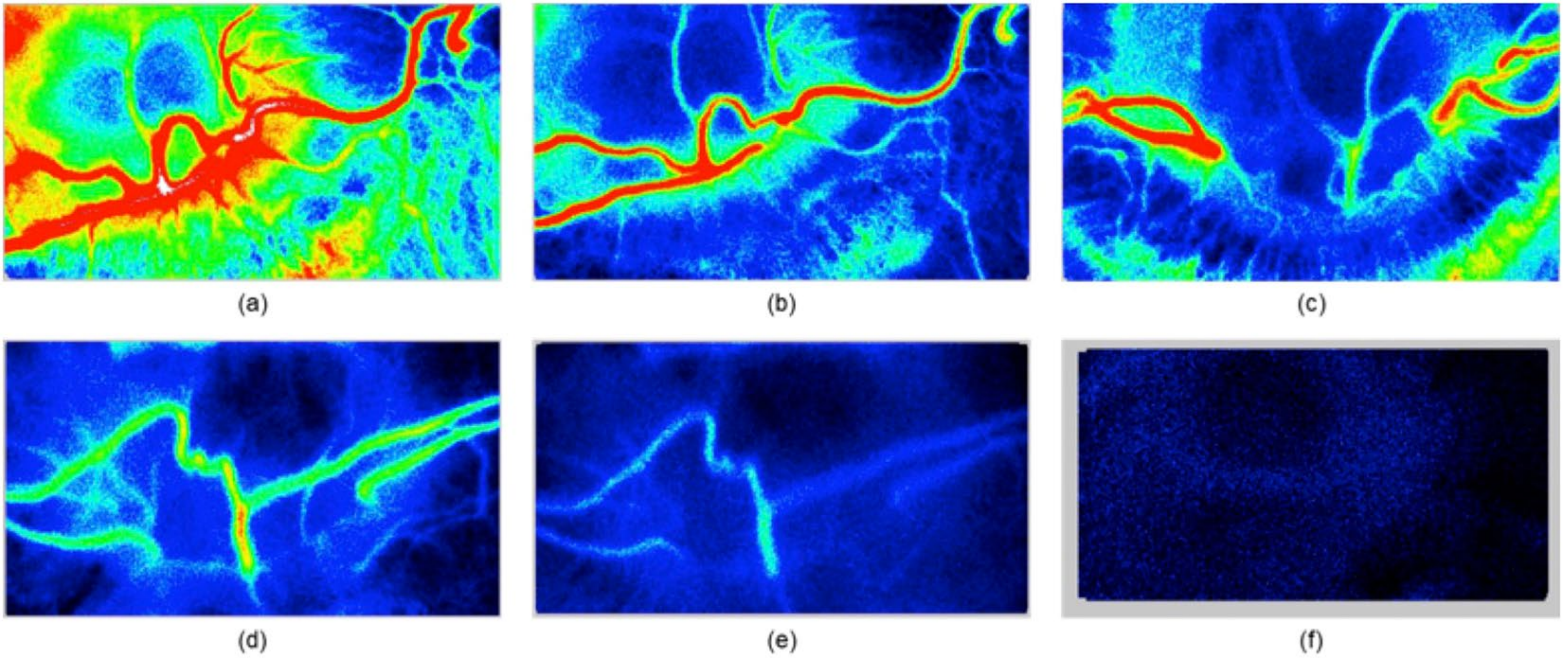
Five different levels of perfusion as noted in the experiment, where (**a**) shows normal perfusion: VPE = 871.1, MV = 28.1, ARBS = 31; (**b**) and (**c**) show limited perfusion in the ONH of two different animals: VPE = 148, MV = 24.8, ARBS = 6 and VPE = 115, MV = 23.1, ARBS = 5, respectively; (**d**) shows weak perfusion: VPE = 59.4, MV = 19.8, ARBS = 3; (**e**) shows diminishing perfusion: VPE = 0, MV = 15.6, ARBS = 0; and (**f**) shows no perfusion at all: VPE = 0, MV = 0, ARBS = 0.

During this study, we encountered five different levels of perfusion, as shown in Fig. 6. All these levels of perfusion were not evidential in all animals. Therefore, the results from different animals are presented in Fig. 6. Figure 6(a) shows normal blood flow in the rabbit’s ONH. Figure 6(b,c) show two ONHs of two different animals and show some perfusion near the vascular regions of the ONHs. Figure 6(d) shows weak perfusion. Figure 6(e) shows almost diminished perfusion. Figure 6(f), which shows no perfusion, was usually recorded minutes before the animal died. In one animal, an image similar to Fig. 6(f) was observed, and on releasing the clip from ECA, the blood flow returned to the normal perfusion level.

We also calculated the perfusion index for the eyes undergoing ECAO. The perfusion index in this case was defined as the ratio of the MBR value in the normal eye before occlusion to the MBR value of the same eye during occlusion. This value was 1.18 ± 0.79 for the vascular part of the entire image, 4.48 ± 7.77 for the tissue part of the entire image, 6.66 ± 10.26 for the MBR of the entire image, and 2.13 ± 1.78 for the MBR of the vascular area subtracted from the MBR of the tissue area.

In three out of the 13 animals used in the second experiment, we performed the experimental protocol on both the eyes. We calculated the LI from all six eyes of these three animals. The LI in this case was defined as the ratio of decreased blood flow due to occlusion in one eye to normal blood flow in its fellow eye. By normal, we mean the baseline value of blood flow without any occlusion. The values of individual eyes are shown in Table 1. The mean LI for the vascular area (MV) was 0.62 ± 0.25, for the tissue area (MT) was 0.40 ± 0.20, for the entire region (MA) was 0.30 ± 0.18, and for the difference between MV and MT (MV-MT) was 0.72 ± 0.33. The mean ARBS value was 0.09 ± 0.12.

**Table 1:** Laterality index as a ratio of decreased blood flow in the occluded eye to the fellow eye (normal) for all the parameters, MV, MT, MA, and MV-MT.

Rabbit	Occluded Eye	MV	MT	MA	MV-MT	ARBS
1	1	1.02	0.58	0.53	1.29	0.31
	2	0.26	0.05	0.03	0.36	0
2	1	0.65	0.31	0.19	0.79	0
	2	0.51	0.43	0.30	0.47	0.01
3	1	0.61	0.57	0.44	0.63	0.17
	2	0.66	0.42	0.26	0.79	0.04

## Discussion

In our study, we showed that the normal non-anesthetized eyes of rabbits depict a strong correlation with their fellow eyes. A similar study demonstrated a significant correlation of blood flow volume in the normal eyes of healthy human subjects^15^. They found the LI for the entire image to be equivalent to one. We have also computed the LI; however, we observed that ocular blood flow in both the eyes is not identical, i.e., one eye always has a slightly higher MBR value than does the other eye. Note that the eye with a higher MBR can be either the right eye or the left eye. We have, therefore, grouped the eyes into the eyes with higher MBR values than the fellow eyes and the eyes with lower MBR values than the fellow eyes. We found the LI for the mean MBR values of the vascular, tissue, MV-MT, and the entire image to be close to one (refer to Table S1).

Our results showed that the MBR values during ECAO decreased significantly to very low or no perfusion level. To the best of our knowledge, this is the first demonstration of real-time recording of ocular blood flow in rabbits under deep anesthesia by applying ECAO and the restoration of blood flow using the LSFG system. LSFG may thus be used as a diagnostic tool for OIS and a monitoring tool during surgical procedures or the recovery phase. We also proposed a new parameter to estimate vascular perfusion and named it the VPE. This parameter shows a significant difference between the normal eyes and the eyes with ECAO, suggesting it is a suitable indicator of successful occlusion/ischemia. For instance, this parameter may in future serve as a biomarker of perfusion in drug efficacy assessment.

We have also calculated another parameter named the LI from six occluded eyes of the three animals subjected to experiment 2 in both the eyes. The LI in this case was defined as the ratio of the MBR value of the occluded eye to the MBR value of the normal eye. The mean value of the tissue and entire area has been found to be less than 0.58 (i.e., the upper limit) for all six eyes. This is in contrast to the value of these parameters observed in normal non-anesthetized animals in which the mean value of all the parameters is greater than 0.76 (lowest limit among all regions of interest). The VPE value after occlusion is also significantly different from the values of this parameter in normal healthy animals.

The decrease in blood flow was in general rapid, and a profound decrease was observed almost instantaneously in few animals. Whilst the decrease in perfusion was not as rapid as in the other animals, all animals showed a very small area of perfusion within 30 minutes of ECAO. Reperfusion in all animals was instantaneous. The effect of ICAO on ocular blood flow was, however, not significant. If the time of ICAO were increased beyond 10 minutes, the systemic parameters would be affected greatly. This affects the ocular blood flow and poses the risk of mortality. Therefore, this study did not determine the effect of a longer duration of ICAO on ocular blood flow in rabbits.

We presented the quantitative false color maps of ECAO wherein five different levels of perfusion were observed. Crudely, perfusion was reduced to complete occlusion in four stages. The first stage showed decreased blood flow in the major vessels, but not a significant decrease. The second stage showed hypoperfusion even in the major vessels. The third stage showed only very small traces of blood flow in the vessels. The fourth stage showed no blood flow. This four-stage ECAO model was achieved without any effect on cerebral perfusion since the ECA of the rabbit does not supply blood to the brain. This model also does not require any induction of high intraocular pressure (IOP), which may exacerbate retinal injury. A previous study showed axonal damage and elevation of the IOP when methods involving ligation of the optic nerve bundle were applied^6^.

The difference in the hemodynamic response to ECAO and ICAO might be explained by how the blood is being supplied to the retina. In the rabbit, the main supply is through the CRA, which might originate from the internal or external ophthalmic artery^17^. The CRA mostly branches from the medial long posterior ciliary artery, but it might also branch from the lateral long posterior ciliary artery, or any of the short posterior ciliary arteries; moreover, it might have more than one root originating from any of the three aforementioned ciliary arteries. These ciliary arteries have several variations in their patterns and all originate from the external ophthalmic artery^17^. In very few animals, the CRA might branch solely from the internal ophthalmic artery^18,19^. In our study, only one rabbit responded to ICAO. The internal ophthalmic artery is a branch of the ICA, whilst the external ophthalmic artery is a branch of the ECA^20^. Our experimental results support the suggestion that the major contribution of retinal blood flow in most of the rabbit eyes come from the ciliary arteries and thus the ECA. The contribution of the ICA was much smaller or less frequent than that of the ECA. To the best of our knowledge, this is the first study that shows *in vivo* measurement of retinal blood flow in rabbits under ECA/ICA ligation followed by reperfusion of these arteries.

Fluorescein angiography (FA) and indocyanine green angiography (ICGA) are two commonly used procedures for detecting retinal ischemia. Both FA and ICGA are invasive tests that necessitate the injection of dyes that might cause comorbidities in patients; therefore, it is not always possible to use these procedures. The time required to complete these procedures is also significantly higher than that required for LSFG measurement (in seconds). LSFG is also preferred over laser Doppler, which measures ocular blood flow. The laser Doppler system is sensitive to probe placement; in contrast, LSFG provides a full-field analysis. Hence, LSFG is more suitable for long-term monitoring of retinal perfusion. Applications of LSFG include monitoring of ocular microcirculation after carotid artery stenting^4^ and during retrograde/antegrade cerebral perfusion^21^. LSFG has been mostly used in patients with glaucoma to assess disease progression^22^. This study for the first time showed the real-time temporal dynamics of occlusion of two major branches of the carotid artery, i.e., the ICA and ECA. This study also demonstrated that LSFG could be used to diagnose occlusion and assess reperfusion in real time.

Magnetic resonance angiography (MRA), computed tomography angiography (CTA), and angiography are common modalities used to detect occlusion in the ECA and ICA^23^. However, these modalities are usually only used after the symptoms of carotid artery disease become evident and the disease has progressed significantly^24^. In addition, CTA poses the risk of radiation exposure and cannot be used for long-term assessment^25^. Furthermore, MRA and CTA facilities are not available in all the hospitals, especially in the developing and least developed countries^26^. Angiography could also pose a risk to patients with kidney dysfunction and comorbidities, especially diabetes^27,28^. Angiography and CTA are invasive procedures that also require the injection of contrast materials and may not be suitable for patients who have had previous reactions to contrast agents, and MRA cannot be used in patients with metallic implants^29,30^. LSFG provides non-invasive, non-contact, real-time imaging of retinal blood flow, and has shown good reproducibility^31,32^. LSFG measurements can be completed within few seconds, and hence, long-term assessment of blood flow is possible using this device.

In short, we conclude that based on LSFG, a non-invasive, non-contact, real-time, and long-term assessment system can be developed to diagnose occlusion in the carotid-ophthalmic pathway. The effects of atherosclerosis on ocular blood flow might be observed longitudinally. We further propose that the parameters LI and VPE can be used as biomarkers to detect hypoperfusion in retinal blood supply. The parameter VPE can be further explored in human studies wherein retinal hypoperfusion is of major concern in eye diseases including, but not limited to, diabetic retinopathy, amaurosis fugax, and neovascular or rubeotic glaucoma.

## Methods

The animal experiments were performed on separate groups of New Zealand white rabbits in order to observe reliable LSFG recordings under different anesthetic regimes, and to assess the effect of vascular occlusion on retinal blood flow. The experiments were approved by the Animal Ethics Committee at Universiti Sains Malaysia (USM/Animal Ethics Approval/2014/(91)(558)), and were conducted in accordance with relevant guidelines and regulations.

### Correlation of blood flow in both eyes

#### Animal preparation

Eight New Zealand white rabbits (only males) were used in this experiment. The rabbits were not anesthetized for this experiment.

#### LSFG recording

Retinal blood flow was monitored using LSFG (Softcare Co., Ltd., Japan), which was equipped with a CCD camera (laser diode, 750 × 360-pixel resolution, 4-second recording at 30 fps). Retinal blood flow was recorded using 10 consecutive LSFG readings per eye in a dark room. All the recordings were carried out with the rabbits lying in the prone position.

### Ocular blood flow changes before, during, and after occlusion

#### Animal preparation

Thirteen New Zealand white rabbits (3 females and 10 males) were used in this experiment. The rabbits were injected with acepromazine (1 mg/0.5 kg) intramuscularly 15 minutes before an intraperitoneal injection of urethane (1200 mg/kg). For all groups, physiological parameters, such as electrocardiogram, heart rate, SpO_2_, end tidal CO_2_, and rectal temperature, were monitored.

#### Experimental protocol

**Figure 7:**
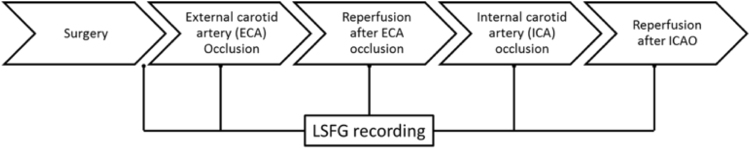
Protocol flow diagram of LSFG recordings before, during, and after occlusions.

Under general anesthesia, a midline incision was made and the left CCA was located and separated from the surrounding tissue. The bifurcation of the CCA was then identified and the ECA and ICA were separated from the surrounding tissue. The ICA was identified as the backward running branch of the CCA with a visible ampulla-like bulge^33^. Two segments of 10-cm-long 2-0 nylon suture were used to keep both arteries separated from the surrounding tissue. The animals were then moved to the stage of the LSFG system. At least 10 consecutive measurements were taken using the accompanying system. These measurements would serve as the baseline measurements. The ECA was then ligated using a surgical clip for 30 minutes, and 10 more measurements were taken. After 30 minutes of occlusion, the artery was allowed to re-perfuse for 10 minutes. If the blood flow returned to ±20% of the baseline value, then the ICA was ligated using a surgical clip and 10 measurements were taken. The clip was removed again 10 minutes after ICAO, and another set of measurements was made. The complete experimental protocol is shown in Fig. 7. Both the eyes of three of the animals were used in the experiment. The physiological conditions of these animals were first checked and returned to normal. Thereafter, the contralateral carotid artery and its bifurcation were exposed before the whole procedure, as shown in Fig. 7, was repeated for the contralateral eye.

#### LSFG recordings

Sixteen eyes of 13 New Zealand white rabbits were subjected to LSFG measurements in a dark room. All the recordings were carried out with the rabbits lying in the prone position.

### Data availability

The datasets generated during and/or analyzed during the current study are available from the corresponding author on reasonable request.

## Electronic supplementary material

Supplementary Information
