# Supplementary material for: Ocular Blood Flow in Rabbits under Deep Anesthesia: A Real-Time Measurement Technique and Its Application in Characterizing Retinal Ischemia
Source: Sci Rep. 2018 Apr 9;8:5713. doi: 10.1038/s41598-018-24141-4 (PMC5890247; doi:10.1038/s41598-018-24141-4)
Supplement: Supplementary file 1 — Supplementary Information [file 41598_2018_24141_MOESM1_ESM.docx]

Ocular Blood Flow in Rabbits under Deep Anesthesia: A Real-Time Measurement Technique and Its Application in Characterizing Retinal Ischemia

Mehwish Saba Bhatti^1^, Tong Boon Tang^1^, Hui Cheng Chen^2^

^1^Centre for Intelligent Signal and Imaging Research (CISIR), Department of Electrical & Electronic Engineering, Universiti Teknologi PETRONAS, Bandar Seri Iskandar 32610, Perak, Malaysia

^2^Department of Companion Animal Medicine and Surgery, Faculty of Veterinary Medicine, Universiti Putra Malaysia, 43400 UPM Serdang, Malaysia.

Corresponding author information

E-mail: tongboon.tang@utp.edu.my

Phone# +60-5-368-7801

Fax# +60-5-368-8386

# Supplementary Information

Table S1. Mean, standard deviation and limits of standard laterality index (LI) values in normal non-anesthetized rabbits.

| **Parameters** | **Mean MBR value** | | **Standard LI value** | | | |
| --- | --- | --- | --- | --- | --- | --- |
|  | **Lower** | **Higher** | **Mean LI** | **Standard Deviation** | **Upper limit** | **Lower limit** |
| MV | 24.70 | 26.19 | 0.94 | 0.02 | 0.99 | 0.89 |
| MT | 7.23 | 7.90 | 0.92 | 0.04 | 0.99 | 0.84 |
| MV-MT | 17.16 | 18.89 | 0.91 | 0.04 | 0.98 | 0.83 |
| MA | 9.89 | 11.60 | 0.86 | 0.05 | 0.95 | 0.76 |


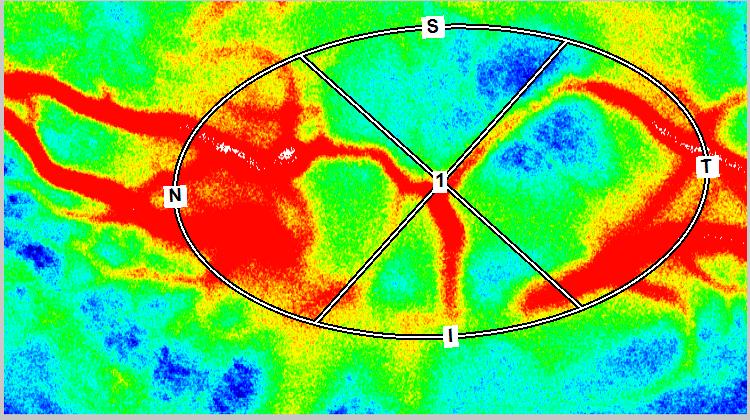


Figure S1. Area of interest at rabbit ONH

A relatively larger area of optic nerve head (ONH) is chosen as another region of interest. This area is further divided into four categories as vascular part (MV-ONH), tissue part (MT-ONH), whole ONH (MA-ONH) and the difference of the mean of vascular and tissue area (MV-MT-ONH). The segmentation of the image into tissue and vascular part is done by manually considering MBR=15 as the threshold value. The results at ONH are shown in Figure S2.


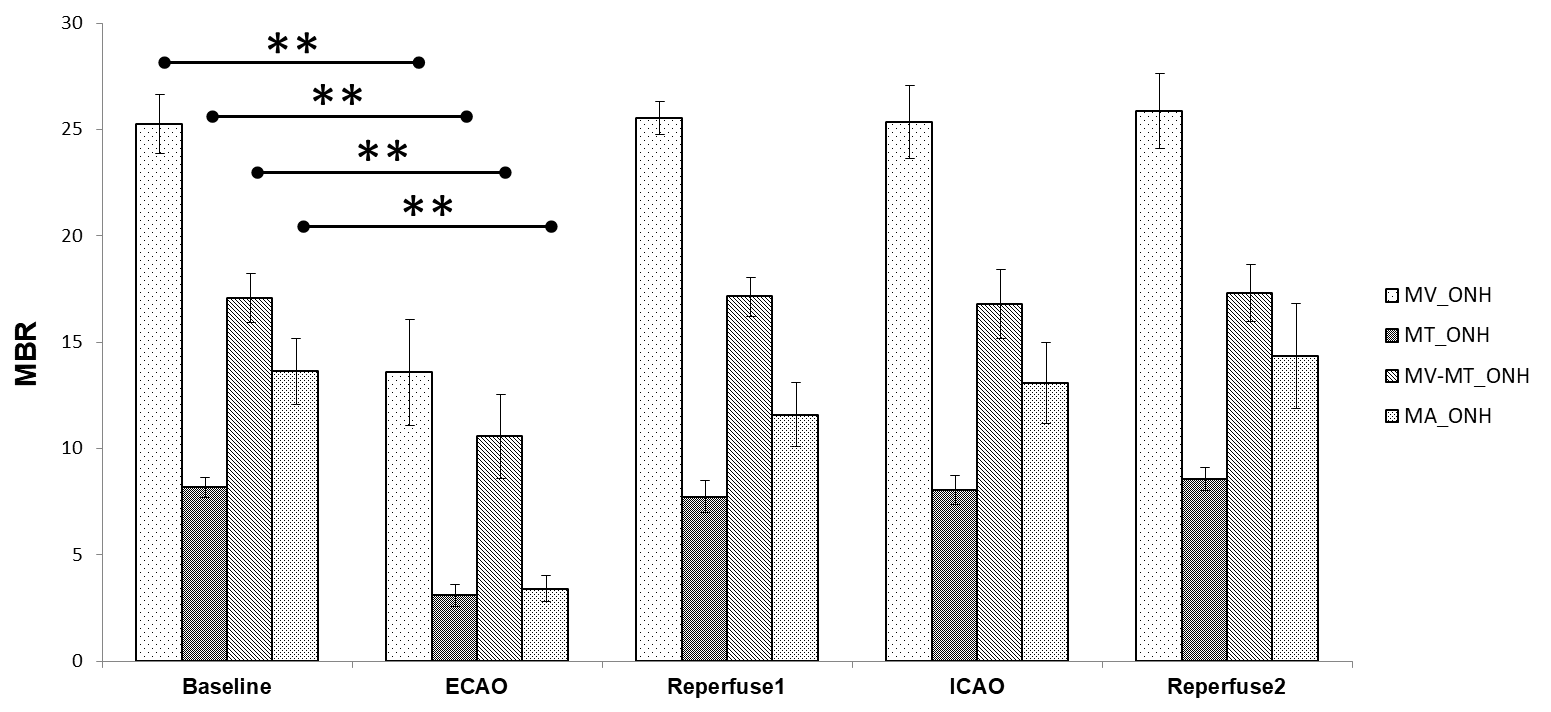


(a)


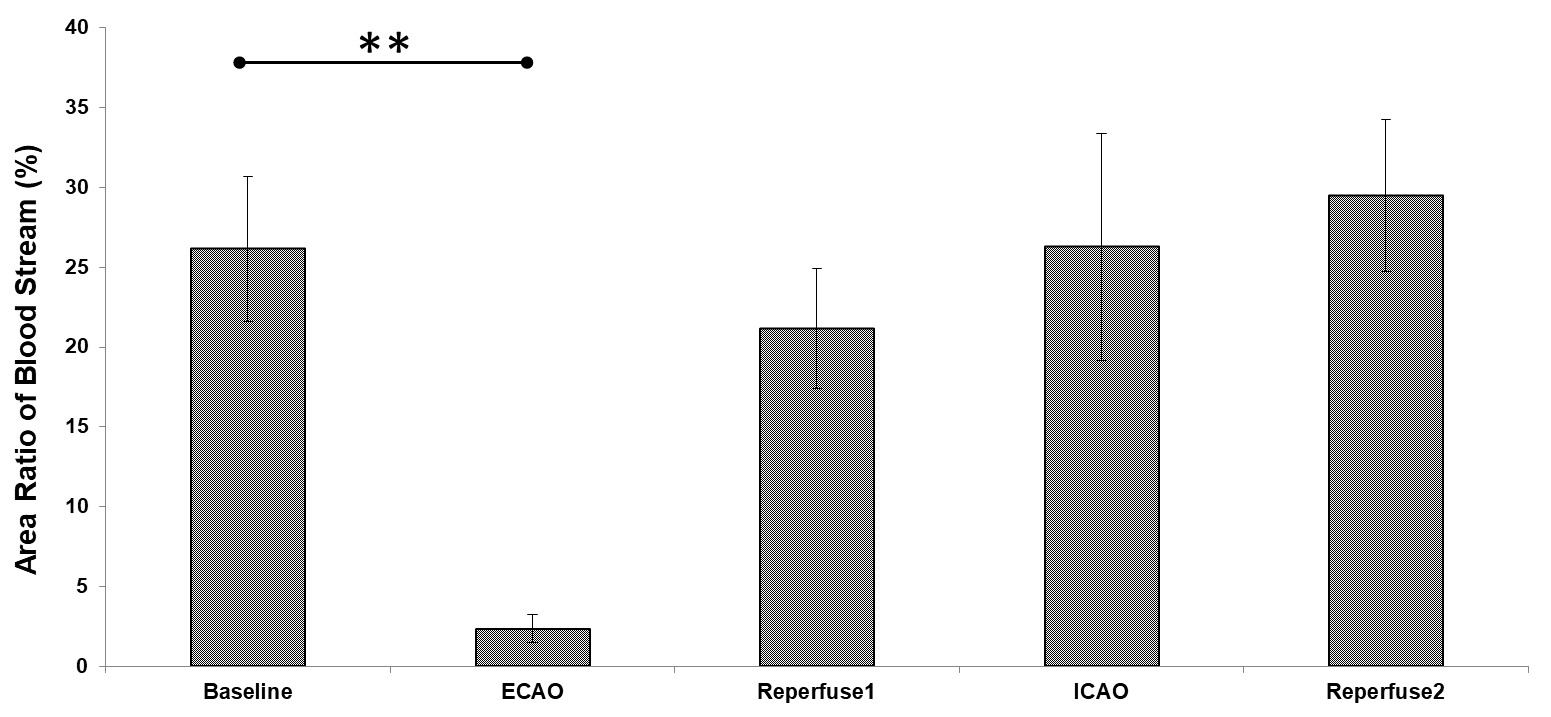


(b)

Figure S2. Results at optic nerve head (ONH) region only: (a) shows the effect of external carotid artery occlusion (ECAO), reperfusion after ECAO (Reperfuse1), internal carotid artery occlusion (ICAO) and reperfusion after ICAO (Reperfuse2), on mean MBR of vascular region (MV-ONH), mean MBR of tissue region (MT-ONH), mean MBR of the whole ONH (MA-ONH) and the mean MBR of vascular area subtracted by tissue area (MV-MT-ONH); (b) shows the area ratio of blood stream during ECAO, reperfusion after ECAO (Reperfuse1), ICAO and reperfusion after ICAO (Reperfuse2). ** signifies p<0.001.
